# To breathe or not to breathe?

**DOI:** 10.7554/eLife.79593

**Published:** 2022-05-20

**Authors:** Lauren C Radlinski, Andreas J Bäumler

**Affiliations:** 1 Department of Medical Microbiology and Immunology, School of Medicine, University of California, Davis Davis United States

**Keywords:** bacterial pathogenesis, cellular respiration, microbial metabolism, Other

## Abstract

*Listeria monocytogenes* uses respiration to sustain a risky fermentative lifestyle during infection.

**Related research article** Rivera-Lugo R, Deng D, Anaya-Sanchez A, Tejedor-Sanz S, Tang E, Reyes Ruiz VM, Smith HB, Titov DV, Sauer JD, Skaar EP, Ajo-Franklin CM, Portnoy DA, Light SH. 2022. *Listeria monocytogenes* requires cellular respiration for NAD+ regeneration and pathogenesis. *eLife* **11**:e75424. doi: 10.7554/eLife.75424.

Bacteria are masters at tuning their metabolism to thrive in diverse environments, including during infection. Constant, life-or-death competition with host immune systems and other microorganisms rewards species that maximize the amount of energy they derive from limited resources.

Living organisms produce energy in the form of a small molecule called adenosine triphosphate (ATP). ATP is generated by breaking down, or oxidizing, high energy molecules such as sugars through a series of electron transfer reactions. These redox (reduction/oxidation) reactions require an intermediate electron carrier such as nicotine adenine dinucleotide (NADH) that must be re-oxidized (NAD^+^) in order for the cell to continue producing ATP by oxidizing high-energy electron donors.

During respiration, NAD^+^ is regenerated when electrons are transferred to a terminal electron acceptor such as oxygen. In organisms that cannot respire, ATP is produced through a less energy-efficient process called fermentation. Fermenting organisms also oxidize high-energy electron donors to produce ATP: however their strategy for regenerating NAD^+^ requires depositing electrons on an organic molecule such as pyruvate. Thus, fermenting organisms sacrifice potential ATP by producing waste products that are not fully oxidized.

*Listeria monocytogenes* is an important foodborne pathogen with an unusual metabolic strategy that falls somewhere between respiration and fermentation. This bacterium carries the genes for two respiratory electron transport chains that can use either oxygen or an extracellular metabolite such as fumarate or iron as a terminal electron acceptor (Corbett et al., 2017; Light et al., 2019). However, unlike most respiring organisms, *L. monocytogenes* lacks the enzymes required to fully oxidize sugars and instead produces partially reduced fermentative end products including lactic and acetic acid (Trivett and Meyer, 1971). Despite this, respiration is absolutely essential for *L. monocytogenes* to cause disease (Corbett et al., 2017) – in fact, mutants that cannot respire are considered safe enough to be used in vaccine development (Stritzker et al., 2004).

Now, in eLife, Samuel Light (University of Chicago) and colleagues – including Rafael Rivera-Lugo and David Deng (both from the University of California at Berkeley) as joint first authors – report on why respiration is essential for an organism that gets its energy through fermentation (Rivera-Lugo et al., 2022).

By comparing the waste products *L. monocytogenes* generates in the presence or absence of oxygen (a terminal electron acceptor), Rivera-Lugo et al. observed that oxygen shifts the composition of *L. monocytogenes* fermentative end products from primarily lactic to exclusively acetic acid. Compared to lactic acid, acetic acid is a slightly more oxidized waste product, the production of which generates more ATP but insufficient NAD^+^ to sustain itself. Thus, while acetic acid production generates more energy, it comes at the cost of redox balance, which could incur a potential reduction in cellular viability. Indeed, Rivera-Lugo et al. observed that genetically disrupting respiratory pathways inhibited *L. monocytogenes* growth within immune cells and prevented the bacterium from causing disease in mice.

Based on these results, Rivera-Lugo et al. surmised that *L. monocytogenes* relies on respiration either to re-oxidize the surplus NADH that results from acetic acid fermentation (and re-establish redox balance), or to generate proton motive force (PMF). PMF is generated when protons are pumped across the bacterial membrane during respiration. The energy stored in the resulting proton gradient can be used by the cell to power important cellular activities such as solute transport and motility.

Redox balance and PMF generation are difficult processes to separate as they involve the same cellular machinery. To determine which of the two is essential for the pathogenesis of *L. monocytogenes*, Rivera-Lugo et al. genetically modified the bacterium to express an unusual NADH oxidase (NOX) previously described in the bacterium *Lactococcus lactis* (Neves et al., 2002). NOX decouples the production of NAD^+^ and PMF by transferring electrons from NADH directly to oxygen without pumping protons across the membrane (Titov et al., 2016). This tool allowed the team to separate these two processes by restoring NAD^+^ regeneration, without increasing PMF. When Rivera-Lugo et al. expressed NOX in a *L. monocytogenes* mutant that cannot respire, NOX activity restored acetic acid production, intracellular growth, cell-to-cell spread, and pathogenesis of the bacterium. This result implies that the primary purpose for *L. monocytogenes* respiration during infection is NAD^+^ regeneration, not PMF production.

While balancing redox may allow *L. monocytogenes* to produce more ATP through the fermentation of acetic acid, it does not explain why a respiration-deficient mutant cannot grow in a host. During infection, *L. monocytogenes* infects and replicates within host cells, then commandeers the cell’s own machinery to spread to neighboring cells. In line with previous observations (Chen et al., 2017), Rivera-Lugo et al. report that genetically inhibiting respiration causes *L. monocytogenes* to lyse – burst open and die – within host cells.

When a bacterium lyses inside a cell, it releases a number of molecules that initiate an antimicrobial response and significantly reduce the bacterium’s ability to cause disease (Sauer et al., 2010). Thus, the tendency for a respiration-deficient mutant to lyse within host cells may inadvertently trigger the host's immune response and lead to clearance of the pathogen from the host. Rivera-Lugo et al. showed that restoring NAD^+^ regeneration with NOX stopped *L. monocytogenes* from lysing within infected cells and restored the bacterium’s ability to colonize a mouse. Together these findings imply that respiration-mediated redox balance is crucial for maintaining *L. monocytogenes* viability during infection (Figure 1).

**Figure 1.**
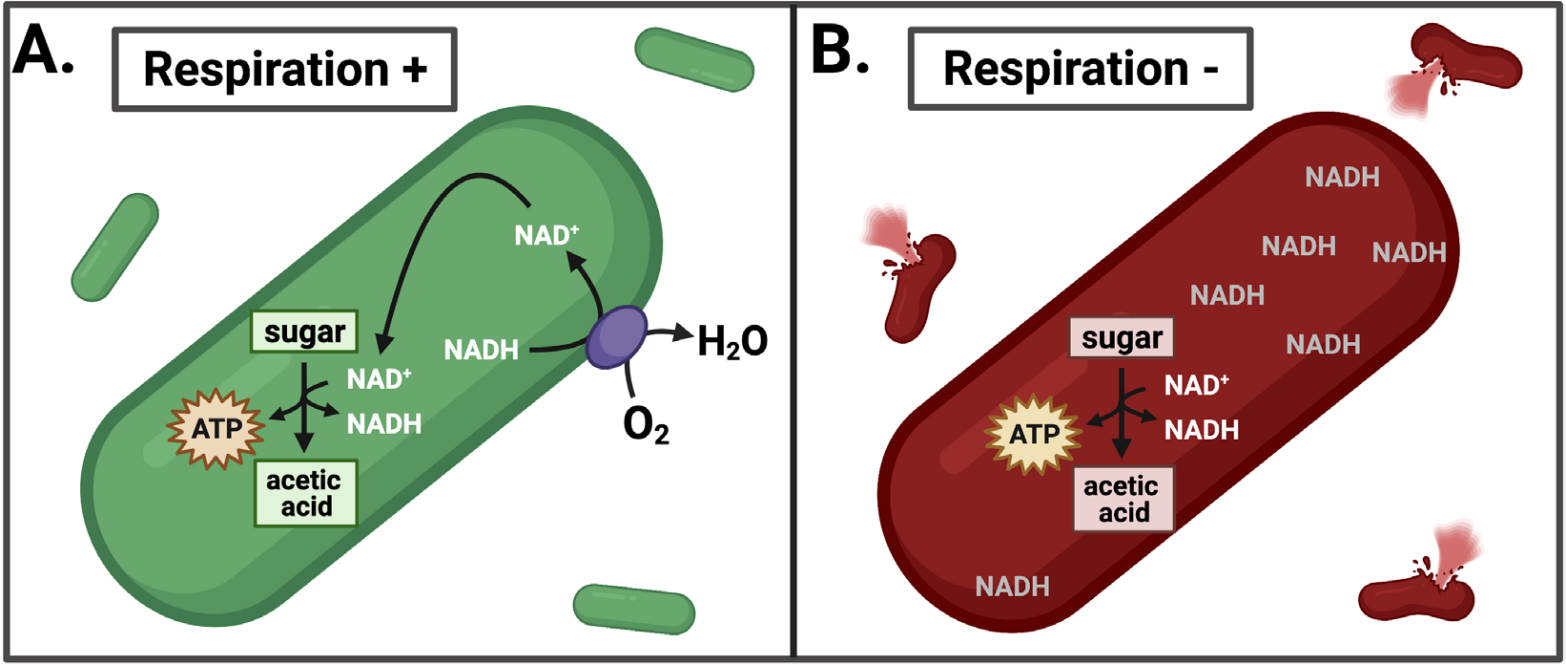
Effects of inhibiting respiration in *L. monocytogenes*. (**A**) *L. monocytogenes* uses respiration to restore redox balance during growth through acetic acid fermentation by transferring electrons from NADH to an electron acceptor such as oxygen (O_2_). This regenerates NAD^+^ to serve as an essential cofactor in the oxidative metabolic reactions that produce ATP. (**B**) Inhibiting respiration causes an imbalance between NAD^+^ and NADH, leading to NADH accumulation and lysis of *L. monocytogenes* during intracellular growth. This leads to a loss of pathogenesis.

The findings of Rivera-Lugo et al. address a long-standing mystery as to why respiration is required for successful *L. monocytogenes* infection. One outstanding question is how the accumulation of NADH leads to lysis. Understanding the molecular mechanism behind this phenomenon, and determining whether inhibiting respiration externally (with a drug, for example) leads to lysis, could reveal new therapeutic approaches for targeting organisms that employ similar respiro-fermentative metabolic strategies during systemic infection.
